# Comparison of Epigenetic Modifier Genes in Bovine Adipose Tissue-Derived Stem Cell Based Embryos, as Donors, with *In Vitro* and Parthenogenesis Embryos

**DOI:** 10.22074/cellj.2020.6714

**Published:** 2019-10-14

**Authors:** Mohammad Salehi, Beheshteh Abouhamzeh, Ahmad Hosseini, Zohreh Zare, Azizollah Bakhtari

**Affiliations:** 1. Cellular and Molecular Biology Research Center, Shahid Beheshti University of Medical Sciences, Tehran, Iran; 2. Department of Biotechnology, School of Advanced Technologies in Medicine, Shahid Beheshti University of Medical Sciences, Tehran, Iran; 3. Department of Anatomical Sciences, Faculty of Medicine, AJA University of Medical Sciences, Tehran, Iran; 4. Department of Anatomical Sciences, Faculty of Medicine, Mazandaran University of Medical Sciences, Sari, Iran; 5. Department of Reproductive Biology, School of Advanced Medical Sciences and Technologies, Shiraz University of Medical Sciences, Shiraz, Iran

**Keywords:** DNA Methyltransferases, Histone Deacetylases, Mesenchymal Stem Cells, POU5F1

## Abstract

**Objective:**

Regarding that undifferentiated mesenchymal stem cells, as donor cells, require less epigenetic reprogramming, possibility of using bovine adipose tissue-derived stem cells (BASCs) with low level of *DNMTs* and *HDACs* expression was evaluated.

**Materials and Methods:**

In this experimental study, we examined gene expression of epigenetic modifiers including DNA methyltransferases (*DNMT1, DNMT3A* and *DNMT3B*) and histone deacetylases (*HDAC1-3*), as well as protein levels of histone H3 acetylation at lysine 9 (H3K9ac) and POU5F1 (also known as OCT4) at two stages of preimplantation development among *in vitro* fertilization (IVF), parthenogenetic activation (PA) and somatic cell nuclear transfer (SCNT) groups.

**Results:**

The results revealed that developmental competence of IVF embryos was higher than SCNT embryos (P<0.05). In the PA and SCNT groups, *DNMT1, HDAC2* and *HDAC3* mRNA were overexpressed (P<0.05), and proteins levels of H3K9ac and POU5F1 were reduced at 6-8 cells and blastocyst stages compared to IVF (P<0.05). The mRNA expression of DNMT1 an<0.05) in both developmental stages (except *HDAC1* in blastocyst stage).

**Conclusion:**

The SCNT embryos derived from BASCs have endured considerable nuclear reprogramming during early embryo development. Comparison of PA and SCNT blastocysts demonstrated that *HDAC1* and *DNMT1* may attribute to developmental competence variability of bovine embryos.

## Introduction

Although several cloned mammals have been created following somatic cell nuclear transfer (SCNT) in a number of animals, less than 5% of them resulted in live birth. One of the reasons for this failure is using differentiated cells as donor cells that are unable to undergo a suitable epigenetic reprogramming, as a necessary point for better developmental competence in the cloned embryos (1).

Abnormal epigenetic marks of DNA and histone, including disruption of imprinted gene expression and high levels of global DNA methylation in the SCNT embryos, are suggested to be the cause of aberrant gene expression during early embryonic development (2). In cloned embryos, global DNA demethylation of CpG sites (except imprinted gene), occurring soon after fertilization, undergoes extensive changes during early embryonic developmental stage (3).

DNA methyltransferase 1 (DNMT1) is responsible for the maintenance of methylation of CpG dinucleotides in the daughter strand of DNA during replication (4). During cleavage-stage, *DNMT1* levels in the nucleus remain low. Nevertheless, DNMT3A and DNMT3B have a pivotal role in de novo methylations which are considerably increased at the 8-16 cells stage in bovine embryos. Unlike bovine IVF-derived embryos, DNA demethylation in the SCNT embryos is not occurred after the 2 cells stage (3). Previous studies have found that using donor cells with low levels of *DNMT1* mRNA for SCNT caused higher developmental competence than those with high levels (5, 6).

Other epigenetic marks of chromatin, including posttranslational modification of histone tails by methylation or acetylation, closely associate with DNA methylation (7). Generally, histone marks are subject to dynamic changes during preimplantation development. In the case of histone H3, lysine acetylation occurs at the lysine sites of 14, 23 18, and 9, in order (8). Acetylation of histone is modulated by histone acetyltransferases (HATs) and deacetylases (HDACs) (9). HDAC also negatively regulates *DNMT1* expression by inhibition of *DNMT1* promoter activity (10).

Studies have shown that Trichostatin A (TSA), a HDAC inhibitor, can improve histone marks in the SCNT embryos derived from various mammalian species. This results in higher developmental competence of the related embryos (2, 11-13). Therefore, it seems necessary to identify normal pattern of histone acetylation to ameliorate potential development of SCNT embryos.

A considerable body of evidences has indicated that success in the SCNT outcome is closely related to the origin of donor cells. As adult stem cells (ASCs), such as mesenchymal stem cells (MSCs), are more differentiated than ESCs, they require additional reprogramming with SCNT (14). Undifferentiated embryonic stem cells (ESCs), as donors, require less epigenetic reprogramming than a differentiated somatic cell, and they show better preimplantation development (15, 16).

Improvement in our understanding of epigenetic reprogramming events will give us insight to the potential of SCNT for various agricultural and biomedical applications. To achieve this goal, bovine adipose derived stem cells (BASCs) were used as donor cells, and dynamic changes of histone H3 acetylation at lysine 9 (H3K9ac) and POU5F1 (also known as OCT4) as well as gene expression of *HDACs* and *DNMTs* were evaluated in two different stages of embryo development in the SCNT, parthenogenetic activation (PA) and *in vitro* fertilization (IVF) derived embryos.

## Materials and Methods

All chemicals and reagents were purchased from Sigma Chemical Co. (USA) and Gibco (USA) unless otherwise speciﬁed

### Oocyte collection and *in vitro* maturation

In this experimental study, local abattoir-derived bovine ovaries were collected and transported to the laboratory at 27-30˚C. Cumulus-oocytes complexes (COCs) were retrieved from antral follicles (3-8 mm). The COCs with several layers of intact cumulus cells and uniformly granulated cytoplasm were selected and cultured in the groups of 10, at 38.5˚C in 50 μl maturation medium tissue culture medium (TCM)-199 supplemented with 10% fetal bovine serum (FBS), 10 ng/ml epidermal growth factor (EGF), 1 μg/ml 17-β estradiol, 10 μg/ml follicle stimulating hormone (FSH), 10 μg/ml luteinizing hormone (LH) and 24.2 mg/l sodium pyruvate) in a humidified 5% CO_2_ for 22-24 hours under mineral oil. Matured oocytes were randomly assigned into three groups, as follows: IVF (n=350), PA (n=443) and SCNT (n=130). All procedures were approved by the Institutional Ethical Committee of the Shahid Beheshti University of Medical Sciences (Tehran, Iran).

### Nuclear donor cell preparation

BASCs, obtained from subcutaneous fat of Holstein cows, immediately after slaughter at a commercial abattoir, were used as nuclear donors. Briefly, fat pieces of 1-2 mm were washed twice in phosphate-buffered saline (PBS) supplemented with 1% penicillin-streptomycin (P/S), and they were digested by 0.5% collagenase type II in 5% CO_2_ at 39˚C for 3 hours in high glucose Dulbecco’s modified Eagle medium (DMEM). Isolated cells were cultured at 39˚C, 5% CO_2_ in DMEM supplemented with 10% FBS, 1% P/S. In order to evaluate differentiation potential, the isolated cells at passage three were treated with osteogenic or adipogenic media. The adipogenic media consisted of DMEM supplemented with 5% FBS, 1% P/S, 0.5 mM isobutyl methylxanthine (IBMX), 250 nΜ dexamethasone and 50 μM indomethacin. Osteogenesis was induced using DMEM with 5% FBS, 1% P/S, 50 μg/ ml L-ascorbic acid biphosphate, 10-7 M dexamethasone and 10 mM beta-glycerophosphate. After 21 days, the cells were fixed in 4% paraformaldehyde solution and stained with alizarin red and oil red for osteogenic and adipogenic differentiation assessment, respectively.

### *In vitro* fertilization, parthenogenetic activation and somatic cell nuclear transfer

The matured oocytes were used for IVF, PA and SCNT. For IVF, groups of 15-20 oocytes were transferred to 100 μl IVF-TALP (Tyrode’s albumin lactate pyruvate) medium containing 114 mM NaCl, 3.2 mM KCl, 0.4 mM NaHPO4, 0.5 mM MgSO_4_, 25 mM NaHCO_3_, 2.6 mM CaCl_2_, 10 mM lactate, 0.25 mM pyruvate, 10 μg/ml P/S, 10 μg/ml heparin and 6 mg/ml bovine serum albumin (BSA). Frozen bull semen was thawed at 37˚C for 30 seconds. The motile spermatozoa were harvested from Percoll gradient (90 and 45% Percoll). Approximately 1×10^6^ sperm/ml were added to IVF-TALP medium containing expanded COCs and co-incubated for 16 hours at 38.5˚C in a humidified atmosphere of 5% CO_2_. Cumulus cells were removed by 1 mg/ml hyaluronidase and vortexing for 4 minutes. The denuded presumptive zygotes were cultured in CR1 medium supplemented with 10% FBS, 2% essential amino acids (EAAs) and 1% nonessential amino acids (NEAAs) at 38.5˚C in a humidified atmosphere of 5% CO_2_, 5% O_2_ and 90% N_2_.

For PA, cumulus cells were removed from mature MII oocytes using 1 mg/ml hyaluronidase and vortexing for 4 minutes. The denuded oocytes were randomly divided into two groups: PA and SCNT. In the PA group, oocytes were incubated in 5 μM Ionomycin for 5 minutes followed by 4 hours exposure to 2 mM 6DMAP in HTCM medium at 38.5˚C and 5% CO_2_ in air. After washing for three times, presumptive parthenotes were cultured in CR1 as described for IVF.

For SCNT, mature denuded oocytes were treated with 0.5 μg/ml demecolcine for 30 minutes and they were placed in the manipulation medium supplemented with 7.5 μg/ml cytochalasin B and subsequently enucleation was performed using a Nikon TE2000U inverted microscope (Nikon, USA) equipped with Narishige micromanipulators at the room temperature. The polar body and MII chromosomes were removed by an 18 ìm (internal diameter) glass pipette. The fifth passage of BASCs with the lowest level of chromatin condensation was used as donor cells for SCNT. A single donor cell was placed into the perivitelline space of each enucleated oocyte through the same hole made previously in the zona pellucida during enucleation. The couplets were electrically fused using two direct current pulses of 150 V/mm for 50 miliseconds in a buffer composed of 0.3 M mannitol, 0.15 mM calcium, 0.15 mM magnesium and 0.01% polyvinyl alcohol (PVA). After one hour, couplets were activated by ionomycin and 6DMAP as described for PA embryos.

For all three groups, cleavage and blastocyst rates were evaluated on day 2 and 8 after insemination or activation, respectively. Seven replicates per group were evaluated. 6-8 cells and blastocysts were collected in order to evaluate *DNMTs* and *HDACs* gene expression using quantitative reverse transcription polymerase chain reaction (qRT-PCR), and perform immunostaining for POU5F1 and H3K9ac.

### RNA extraction, cDNA synthesis and quantitative reverse transcription polymerase chain reaction

Embryos at the 6-8 cells (embryonic genome activation) and blastocyst stages of development, in all groups (IVF, PA and SCNT) were removed from CR1 medium and washed in PBS. RNA extraction was performed as previously described (17). Briefly, 30 embryos in three biological replicates (10 embryos for each replicate per group) were transferred into 0.2 ml nuclease-free tubes containing 1.5 μl lysis buffer plus 2 μl poly N and 5 μl nuclease free water. The tubes were placed in a Thermal Cycler (Applied Bio-Rad, USA) at 75˚C for 5 minutes, followed by adding 5 μl RT Buffer (5X), 1 μl RT Enzyme (200 U), 3 μl dNTP (10 mM) and 0.25 μl RNase inhibitor (10 U) to each tube. cDNA synthesis program for each embryo pool (6-8 cells and blastocyst) was as follows: 25˚C for 10 minutes, 37˚C for 15 minutes, 42˚C for 45 minutes and 72˚C for 10 minutes.

In order to evaluate gene expression pattern of *DNMTs (DNMT1, DNMT3A* and *DNMT3B*) and HDACs (*HDAC1, HDAC2* and *HDAC3*) at two stages of preimplantation development, qRT-PCR was performed in the Rotor Gene Q instrument (Qiagen, Germany). PCR reaction was performed in a final volume of 13 μl consisting of 6.5 μl of 2X SYBR Green master mix (Quanta, USA) and 1 μl mixed primer (10 pmol/μl), and 1 μl cDNA. At least three biological replicates were used for each group. *GAPDH* was used as a reference gene for normalization of the comparisons within the same developmental stage. Relative gene expression was then calculated using the 2^-ΔΔCt^ method (18). The primers used for qRT-PCR are listed in Table 1.

**Table 1:** Details of primers used for quantitative reverse transcription polymerase chain reaction


Gene	Nucleotide sequences (5′–3′)	Fragment size (bp)	Accession number

DNMT1	F: CGGAACTTCGTCTCCTTC	114	NM_182651.2
	R: CACGCCGTACTGACCAG		
DNMT3A	F: TTACACAGAAGCATATCCAGG	143	NM_001206502.1
	R: GAGGCGGTAGAACTCAAAG		
DNMT3B	F: ATCTTGTGTCGTGTGGGG	140	NM_181813.2
	R: CTCGGAGAACTTGCCATC		
HDAC1	F: AGAGAAGAAAGAAGTCACAGAAG	135	NM_001037444.2
	R: GGATAAAGGTAGGGATTTGG		
HDAC2	F: GGCGGTCGTAGAAATGTG	162	NM_001075146.1
	R: TTCTGATTTGGCTCCTTTG		
HDAC3	F: GATGACCAGAGTTACAAGCAC	193	NM_001206243.1
	R: CCAGTAGAGGGATATTGAAGC		
GAPDH	F: GTCGGAGTGAACGGATTC	176	NM_001034034.2
	R: TTCTCTGCCTTGACTGTGC		


### Immunofluorescent staining of embryos

Presence of POU5F1 and H3K9ac was assessed by immunofluorescence staining at two stages of preimplantation development (6-8 cells and blastocyst), as previously described (19). Briefly, embryos were washed in washing buffer (PBS- containing 0.1% Tween-20 and 1% BSA), and ﬁxed for 20 minutes in 4% paraformaldehyde. After three times washing, the embryos were permeabilized with 0.5% Triton X-100 in washing buffer for 40 minutes and incubated with blocking buffer containing 0.1% Triton X-100 and 10% normal goat serum in washing buffer for 60 minutes. The embryos were then incubated in either rabbit polyclonal anti H3K9ac (1:200; Abcam, UK) or rabbit polyclonal anti OCT4 antibody (1:200, Abcam, UK) in blocking buffer overnight at 4˚C. After several times washing, the embryos were incubated in goat anti-rabbit IgG ﬂuorescein conjugated (1:500, Abcam, UK) for 60 minutes. Following DNA staining by 1 μg/ ml 4,6-diamino-2-phenylindole (DAPI), the embryos were mounted on slides, and imaged by ﬂuorescence microscope (Olympus, Japan). Images were quantified by ImageJ software (v. 1.48, National Institute of Mental Health, USA). Briefly, the average gray value was measured by manually outlining the nuclear intensity of blastomeres and corrected based on the mean gray value of five different cytoplasmic areas, as a background.

### Statistical analysis

Normality of data was evaluated, and all data was verified for homogeneity of variances by Levene’s Test. Data were analyzed by one-way ANOVA as well as duncan’s post-hoc test for multiple comparison of groups, using IBM SPSS statistics for windows, version 20.0 (SPSS Inc. Chicago, IL, USA). P<0.05 were considered statistically significant.

## Results

### Nuclear donor cell preparation

Multipotent differentiation potential of BASCs was verified by differentiation into the osteogenic and adipogenic lineages. DNA methyltransferases (*DNMT1, DNMT3A* and *DNMT3B*) and histone deacetylases (*HDAC1, HDAC2* and *HDAC3*) mRNA expression were evaluated at the third, fifth and seventh passages. The results indicated that *DNMTs* and *HDACs* were significantly downregulated at the fifth passage (P<0.05). The highest levels of H3K9ac and POU5F1 were also detected at this passage (P<0.05). Regarding the upregulation of stemness and downregulation of chromatin condensation at the fifth passage, the cells at this passage were considered as donor cells for SCNT.

### Effect of different *in vitro* embryo production procedures on developmental competence

Nine hundred and twenty-three bovine oocytes in seven replicates were matured and randomly divided into three groups of IVF, PA and SCNT. As shown in Table 2, the rate of embryo cleavage among the three groups was not significantly different. The blastocyst development rate in the IVF and PA groups (39.11 ± 2.36 and 34.41 ± 3.54 for IVF and PA groups, respectively) was significantly higher (P<0.001) than SCNT group (14.19 ± 2.43).

### Expression of *DNMTs* and *HDACs* in bovine preimplantation embryos derived from IVF, PA and SCNT

**Fig 1:**
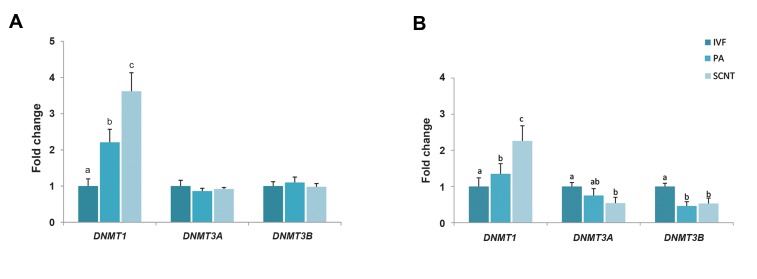
Analysis of DNA methyltransferase gene expression among the three groups. Transcript abundance of *DNMT1, DNMT3A* and *DNMT3B* at the **A.** 6-8 cells and **B.** Blastocyst stages in bovine embryos derived from IVF, PA and SCNT. Different superscripts (a, b, c) indicate a significant difference between groups (P<0.05). Data are expressed as mean ± standard error mean (SEM). IVF; *In vitro* fertilization, PA; Parthenogenetic activation, and SCNT; Somatic cell nuclear transfer.

Transcript abundance of DNA methyltransferases (*DNMT1, DNMT3A* and *DNMT3B*) and histone deacetylases (*HDAC1, HDAC2* and *HDAC3*) was evaluated for each group at the 6-8 cells and blastocyst stages. The highest and lowest level of *DNMT1* transcript was found at both stages of 6-8 cell and blastocyst in the respectively SCNT and IVF groups (P<0.05, Fig .1A, B).

Although there was no significant difference between these groups for the expression level of *DNMT3A* and *DNMT3B* at the 6-8 cells stage, the expression level was significantly lower in SCNT than IVF group at blastocyst stage. In addition, *DNMT3B* mRNA level was lower in PA group compared to IVF group at blastocyst stage (P<0.05, Fig .1B).

**Table 2:** Effect of three different bovine embryo production on developmental competence


Group	Number of oocytes	Cleavage (% ± SEM)	8-16 cells (% ± SEM)	Blastocyst (% ± SEM)

IVF	350	73.69 ± 2.88	46.72 ± 2.19^a^	39.11 ± 2.36^a^
PA	443	81.09 ± 2.96	47.01 ± 4.49^a^	34.41 ± 3.54^a^
SCNT	130	77.34 ± 4.70	35.67 ± 3.02^b^	14.19 ± 2.43^b^


IVF; *In vitro* fertilization, PA; Parthenogenetic activation, SCNT; Somatic cell nuclear transfer, and ^a, b^; Within each column, superscript letters represent statistically signi.cant differences between groups (P<0.05).

**Fig 2:**
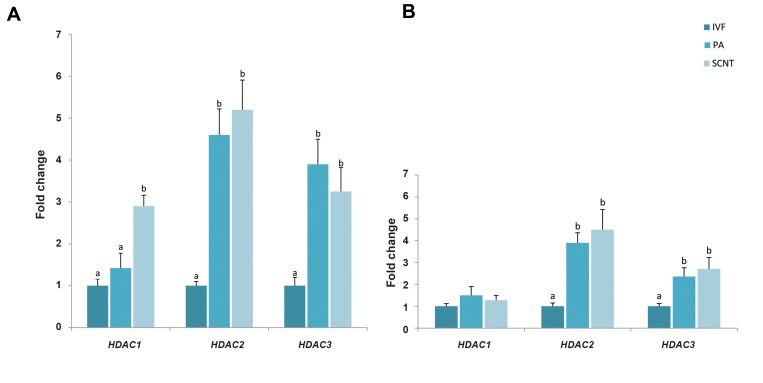
Analysis of histone deacetylase gene expression among the three groups. Transcript abundance of *HDAC1, HDAC2* and *HDAC3* at the **A.** 6-8 cells and **B.** Blastocyst stages in bovine embryos derived from IVF, PA and SCNT. Different superscripts (a, b) indicate a significant difference between the groups (P<0.05). Data are expressed as mean ± SEM. IVF; In vitro fertilization, PA; Parthenogenetic activation, and SCNT; Somatic cell nuclear transfer.

We found the highest level of *HDAC1* in SCNT embryos at the 6-8 cells stage (P<0.05, Fig .2A), not the blastocyst stage (Fig .2B). In PA and SCNT groups, the expression level of *HDAC2* and *HDAC3* was higher than IVF group at the 6-8 cells and blastocyst stages (P<0.05, Fig .2A, B).

### Effect of *in vitro* embryo production on POU5F1 and acetylation of H3K9 in bovine embryos

**Fig 3:**
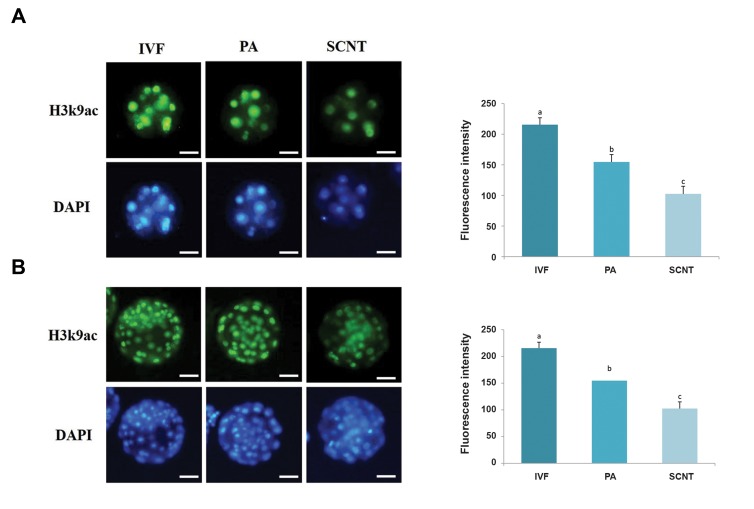
Comparison of H3K9ac .uorescence intensity among the three groups. Immunofluorescent staining of H3K9ac at the **A.** 6-8 cells and **B.** Blastocyst stages in bovine embryos derived from IVF, PA and SCNT. Different superscripts (a, b, c) indicate a significant difference between groups (P<0.05) (scale bar: 50 μm). Data are expressed as mean ± SEM.. IVF; *In vitro* fertilization, PA; Parthenogenetic activation, and SCNT; Somatic cell nuclear transfer.

**Fig 4:**
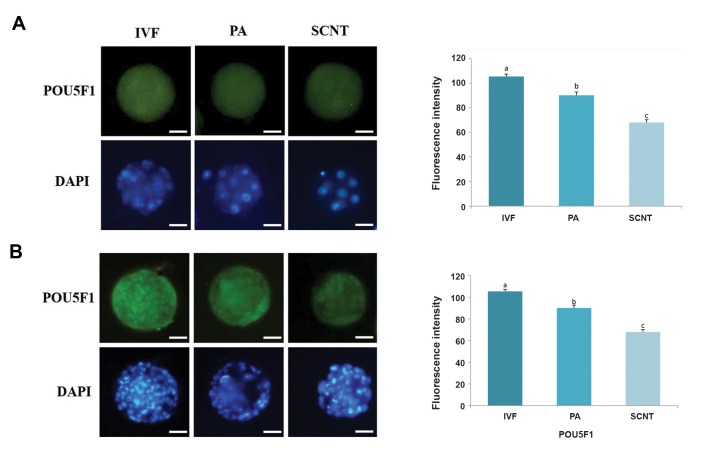
Comparison of POU5F1 ﬂuorescence intensity among the three groups. Immunofluorescent staining of POU5F1 at the **A.** 6-8 cells and **B.** Blastocyst stages in bovine embryos derived from IVF, PA and SCNT. Different superscripts (a, b, c) indicate a significant difference between the groups (P<0.05) (scale bar: 50 μm). Data are expressed as mean ± SEM. IVF; *In vitro* fertilization, PA; Parthenogenetic activation, and SCNT; Somatic cell nuclear transfer.

The .uorescence intensity of H3K9ac and POU5F1 were not significantly different between inner cell mass (ICM) and trophectoderm (TE, data not shown). Thus, ICM and TE blastomers, both were used to evaluate the .uorescence intensity. Figures 3 and 4 reveal the H3K9ac and POU5F1 protein contents in the experimental groups at two different stages of preimplantation development. In the SCNT group, the .uorescence intensity of H3K9ac and POU5F1 were lower than the IVF and PA groups in both the 6-8 cells and blastocyst stages (P<0.05). Additionally, there was statistically significant difference between PA and IVF groups (P<0.05).

## Discussion

Results of this study showed a reduction in the blastocyst rate of SCNT group with high levels of *DNMT1* and *HDAC2-3* transcript abundance and less fluorescence intensity of H3K9ac and POU5F1 at the 6-8 cells and blastocyst stages in comparison with IVF group. *DNMT3A* and *B* mRNA level was low at blastocyst stage and HDAC1 was high at the 6-8 cells stage in SCNT group compared to IVF group. Data also indicated an increasing in the level of *DNMT1* and *HDAC2-3* and a reduction in the .uorescence intensity of H3K9ac and POU5F1 in PA embryos at the 6-8 cells and blastocyst stages.

Since the creation of Dolly as the first successful SCNT in sheep, various somatic cells have been used as donors for SCNT (20, 21). Previous studies have demonstrated that the differentiation and methylation state of donor cells can affect efficiency of SCNT process (1, 22). Based on the previous studies, showing that donor cells with low levels of *DNMT1* mRNA as well as inhibition of HDACs could improve developmental competence in the SCNT embryos (2, 5, 6, 11, 13), in the present study, for first time, BASCs were used as donor cell at passage five with the lowest levels of *DNMTs (DNMT1, DNMT3A* and *DNMT3B)* and *HDACs (HDAC1, HDAC2* and *HDAC3)* genes.

A considerable body of evidences indicates the incomplete demethylation and early remethylation of cloned embryos derived by SCNT (3, 23). Thus, we conducted this study aiming to investigate whether using a donor cells with low mRNA levels of DNA methyltransferases (*DNMT1, DNMT3A* and *DNMT3B*) would modulate epigenetic status of resultant cloned embryos at the 6-8 cells and blastocyst stages. Our findings revealed that at the 6-8 cells embryos, the *DNMT1* mRNA level was more than three-times greater in the SCNT group compared to IVF group. Moreover, embryos derived from PA, which almost were female, showed higher mRNA levels of *DNMT1* compared to IVF group. This pattern of gene expression was also maintained by the blastocyst stage.

*De novo* methyltransferases were evaluated in the three groups and data showed similar level of expression for both *DNMT3A* and *DNMT3B* mRNA at the 6-8 cells embryos which is consistent with Golding et al. (24) study. At the blastocyst stage, however, *DNMT3A* and *DNMT3B* expressions in the SCNT group were lower than IVF group. This is in contrast with the previous study that did not find any significant difference in the de novo DNA methyltransferases expression among the three groups of IVF, PA and SCNT. In another study performed by Wang et al. (25), *Dnmt3a* expression was found to be lower in bovine SCNT blastocysts compared to IVF, whereas the *Dnmt3b* was higher in the SCNT group versus IVF. These differences between different studies might be related to the epigenetic status, differentiation level of donor cells, and/or *in vitro* culture conditions. Bakhtari and Ross (26) suggested that protection of at least one pronucleus from DNA demethylation may be required for normal preimplantation development. On the other hand, another study reported that treatment of donor cells with 5-aza-20-deoxycytidine (5-aza-dC), a DNA methylation inhibitor, could not ameliorate developmental competence of bovine SCNT embryos (27). In contrast, treatment of early SCNT embryos with TSA, a *HDAC* inhibitor, showed similar blastocyst rate compared to IVF group (11, 27). Therefore, we proposed that histone reprogramming might be more important than establishment of normal DNA modification in the cloned embryos.

*HDACs* are commonly expressed in the various tissues and different stages of preimplantation development and they play a pivotal role in the modulation of gene expression (28). In our study, *HDAC1-3* expression levels were compared among IVF, PA and SCNT groups during 6-8 cells and blastocyst stages. The results showed that *HDAC1* mRNA level at the 6-8 cells, but not at the blastocyst stage, was significantly affected by SCNT process. Transcript abundance of both *HDAC2* and *HDAC3* was higher in the PA and SCNT groups at the 6-8 cells and blastocyst stages in comparison with IVF. Beyhan et al. (29) indicated that increasing mRNA level of *HDACs* in the SCNT embryos at the morula stage might be required to promote transcriptional silencing in order to reprogram the somatic cells nuclei. However, in our study, *HDAC1* mRNA level in the SCNT blastocysts is comparable to IVF and PA groups. Ma and Schultz (30) demonstrated that *Hdac1* is the main *Hdac* transcript in the preimplantation of mouse embryos. On the other hand, in our experiment, the PA group which had a normal *HDAC1* expression level showed the same blastocyst formation rate compared to IVF group. Thus, it seems that *HDAC1* is more important than *HDAC2* and *HDAC3* in preimplantation development of bovine embryos. In this study, one reason for the similar level of *HDAC1* expression among these three groups and reduction of blastocyst rate in the SCNT group might be due to undesirable effects of the *HDAC1* transcript overexpression for the normal development up to blastocyst stage. Thus, regulation of *HDAC1* transcription may play an important role in improvement of bovine SCNT embryo developmental competence. It is likely that SCNT blastocysts were rescued from gene suppressing, by *HDAC1*. Previous study demonstrated that DNMT1 is associated with histone deacetylase activity and it has a transcriptional repressor domain which can interact with the *HDAC1* (31). Suppression of the DNMT1 expression in the 6-8 cells stage embryos might be due to the upregulated expression of *HDAC1*.

Acetylation of histone tail residues is an important process resulting in chromatin unfolding and allow access to the regulatory transcriptional factors (32). In this study, we assessed the rate of H3K9ac during 6-8 cells and blastocyst stages, using immunofluorescence staining. Our finding showed that the value of acetylated H3K9 in the SCNT group was less than IVF and PA groups. Regarding the impact of HDACs on the acetylation of H3K9 (33), reduction of H3K9ac in the PA and SCNT groups might be resulted from overexpression of HDACs.

Since *Pou5f1* is the earliest expressed gene to encode a transcription factor in mouse embryos, and it plays a critical role in the self-renewal of undifferentiated embryonic stem cells (34), we evaluated the level of this protein at the 6-8 cells and blastocyst stages. Results showed that in the SCNT group, this level was less than that of the other groups. In addition, a significant difference of *POU5F1* was found between IVF and PA groups which is consistent with the previous study indicating that the *POU5F1* and *DNMT3A* genes were downregulated in the PA (35) and SCNT embryos (29) versus IVF embryos. It has been demonstrated that HDAC1 and HDAC2 as a multiprotein complex are associated with *POU5F1* gene expression (36). Thus, *HDAC1-2* overexpression may result in reduction of *POU5F1* expression level in the resultant embryos.

## Conclusion

The results of this study demonstrated that i. The rate of blastocyst formation in the cloned bovine SCNT embryos derived from BASCs with low mRNA levels of *DNMT1* and *HDACs* (except *HDAC1* in blastocyst stage), was less than that of the IVF group, ii. Different values of H3K9ac and POU5F1, detected among the groups and over the different developmental stages, may be related to the overexpression of HDACs in the PA and SCNT groups, and iii. Despite various aberrant epigenetic modifications in preimplantation development of both PA and SCNT groups, normal blastocyst rate of the PA compared to SCNT embryos may be related to the improving role of *HDAC1* and *DNMT1* in the developmental competence of bovine embryos.
